# Heart rate variability as a function of menopausal status, menstrual cycle phase, and estradiol level

**DOI:** 10.14814/phy2.15298

**Published:** 2022-05-24

**Authors:** Sharanya Ramesh, Matthew T. James, Jayna M. Holroyd‐Leduc, Stephen B. Wilton, Darlene Y Sola, Sofia B. Ahmed

**Affiliations:** ^1^ Temerty Faculty of Medicine University of Toronto Toronto Ontario Canada; ^2^ Cumming School of Medicine University of Calgary Calgary Alberta Canada; ^3^ Libin Cardiovascular Institute Calgary Alberta Canada

**Keywords:** heart rate variability, women, Estrogen, Menopause, menstrual cycle, female

## Abstract

Low estradiol status is associated with increased cardiovascular risk. We sought to determine the association between heart rate variability (HRV), a marker of cardiovascular risk, at baseline and in response to stressor as a function of menopausal status, menstrual cycle phase and estradiol level. Forty‐one healthy women (13 postmenopausal, 28 premenopausal) were studied. Eleven premenopausal women were additionally studied in the high and low estradiol phases of the menstrual cycle. HRV was calculated by spectral power analysis (low Frequency (LF), high frequency (HF) and LF:HF) at baseline and in response to graded Angiotensin II (AngII) infusion. The primary outcomes were differences in HRV at baseline and in response to AngII. Compared to premenopausal women in the low estradiol phase, postmenopausal women demonstrated lower baseline LF (*p* = 0.01) and HF (*p* < 0.001) measures, which were not significant after adjustment for age and BMI. In response to AngII, a decrease in cardioprotective HRV (ΔHF = −0.43 ± 0.46 ln ms^2^, *p* = 0.005 vs. baseline) was observed in postmenopausal women versus premenopausal women. Baseline HRV parameters did not differ by menstrual phase in premenopausal women. During the low estradiol phase, no differences were observed in the HRV response to AngII challenge. In contrast, women in the high estradiol phase were unable to maintain HRV (ΔLF = −0.07 ± 0.46 ln ms^2^, *p* = 0.048 response vs. baseline, ΔHF = −0.33 ± 0.74 ln ms2, *p* = 0.048 response vs. baseline). No association was observed between any measure of HRV and estradiol level. Menopausal status and the high estradiol phase in premenopausal women were associated with reduced HRV, a marker of cardiovascular risk. Understanding the role of estradiol in the modulation of cardiac autonomic tone may help guide risk reduction strategies in women.

## Graphical abstract

We sought to determine the association between heart rate variability, a marker of cardiovascular risk, at baseline and in response to stressor as a function of menopausal status, menstrual cycle phase, and estradiol level. Our results show that menopausal status and the high estradiol phase in premenopausal women were associated with reduced heart rate variability. Understanding the role of estradiol in the modulation of cardiac autonomic tone may help guide risk reduction strategies in women.

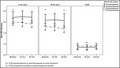

## 1 INTRODUCTION

Women are relatively protected against cardiovascular disease (CVD) until menopause, after which the CVD risk increases significantly to match or exceed the risk in men (Rosano et al., 2007). Despite overall improvements in CV care, young individuals, and especially women, continue to show much slower reductions in CVD mortality (Wilmot et al., 2015). Menopause at a younger age is associated with increased CVD risk (Kannel et al., 1976; Muka et al., 2016) suggesting that early loss of endogenous estradiol may be the important factor in early‐onset CVD (Ossewaarde et al., 2005; Rocca et al., 2006), though the mechanism is not clear. Controversy exists as to the role of exogenous estradiol in the prevention of CVD (Anderson et al., 2004; Hodis et al., 2016; Manson et al., 2013; Miller et al., 2019; Rossouw et al., 2002), underscoring the importance of understanding how endogenous estradiol affects measures of CV risk.

Heart rate variability (HRV) is a validated marker of CVD risk (Camm et al., 1996) in both high (Drawz et al., 2013; Kleiger et al., 1987; Silva‐E‐Oliveira et al., 2017) and low‐risk healthy populations (Soares‐Miranda et al., 2014; Tsuji et al., 1994). Previous studies have examined HRV based on menopausal status (Gökçe et al., 2005; Liu et al., 2003; Moodithaya & Avadhany, 2009; Virtanen et al., 2015; Yang et al., 2013) and throughout the menstrual cycle (Matsumoto et al., 2007; Princi et al., 2005; Sato & Miyake, 2004; Vallejo et al., 2005; Zambotti et al., 2013), with conflicting observations that may be attributed to variable use of exogenous estradiol therapies, alterations in dietary salt intake, adiposity, or time of measurement, all of which can affect HRV (Babcock et al., 2018; Kiselev et al., 2018; Sammito et al., 2016; Schmalenberger et al., 2019, 2020; Windham et al., 2012). The effect of menopausal and menstrual status, and endogenous estradiol specifically, on HRV has not been fully evaluated. We sought to determine the effect of menopausal status, menstrual cycle phase, and endogenous estradiol on HRV as a measure of CV risk in postmenopausal and premenopausal women throughout the menstrual cycle, both at baseline and in response to a physiologic stressor.

## 2 METHODS

### 2.1 Participants

Forty‐one self‐identified women with female sex assigned at birth (13postmenopausal, 28 premenopausal) who were healthy, normotensive, and non‐smokers were enrolled. The University of Calgary Conjoint Health Research Ethics Board approved the study and each participant provided written informed consent. Participants underwent a detailed history and physical examination. None were on regular medications including postmenopausal hormone therapy or systemic hormonal contraception. To account for variations in sex hormone levels and renin–angiotensin aldosterone system (RAAS) activity through the menstrual cycle (Chidambaram et al., 2002), premenopausal women were studied 14 days after the 1st day of the menstrual cycle (high estradiol phase) and 11 participants were studied again on the 1st day of the menstrual cycle (low estradiol phase). Both phases were verified by measuring serum sex hormone levels.

All participants were counseled to adhere to a diet that maintained their normal daily caloric intake while maintaining a high‐salt state (150 mmol/day) for 3 days prior to the study day to ensure maximal RAAS suppression (Shoback et al., 1983). Compliance with the high‐salt diet was verified by urine collection. Each study commenced at 0800 h following an overnight fast in a quiet, temperature‐controlled room with participants in a supine position.

### 2.2 Study protocol

An 18‐gauge peripheral venous cannula was inserted into each antecubital vein for infusion and blood sampling. Systolic blood pressure (SBP), diastolic blood pressure (DBP), and mean arterial pressure (MAP) were collected via an automated sphygmomanometer (Dinamap, GE Healthcare, Waukesha WI) every 15 minutes. Participants were infused with graded, incremental doses of Ang II (3 ng/kg/min ×30min; 6 ng/kg/min ×30min), followed by a 30 min recovery period.

### 2.3 Heart rate variability measurements

Ambulatory ECG data were collected with a commercially available Holter monitor (SEER MC Recorder, GE Healthcare). Holter data were collected continuously for 180 min (baseline (30 min), each dose of Ang II (time 30 min, 3 ng/kg/min and time 60 min, 6 ng/kg/min), and recovery (30 min). Frequency domain measures of heart rate variability (HRV) were calculated using standard methods (MARS version 7, GE Healthcare) (Kannel et al., 1976). Frequency domain parameters were derived using power spectral analysis, where cardiosympathetic power was partially represented by the low‐frequency (LF; ms^2^) band within the range of 0.04–0.15 Hz, and cardiovagal power was represented by the high‐frequency (HF; ms^2^) band within the range of 0.15–0.40 Hz. The LF:HF ratio component was calculated by comparing non‐normalized, absolute LF, and HF parameters.

### 2.4 Analytical methods

Plasma estradiol, progesterone, and testosterone levels were determined using radioimmunoassay (Roche E170 Modular, Roche Diagnostics). The lower limit of detection for the measurement of plasma progesterone is <1 nmol/L. As such, we assigned a value of 0.99 nmol/L for these data points. Plasma renin activity (PRA) was determined by the quantification of plasma Angiotensin I, a surrogate measure of PRA, by radioimmunoassay (PRA 125I, DiaSorin). Ang II plasma levels were measured by standard laboratory immunoassay techniques (Quest Diagnostics). Serum cholesterol assays were quantified by enzymatic colorimetric assay techniques (Roche/Hitachi Creatinine Plus; CHOD‐PAP, HDL‐C Plus, and TG GPO‐PAP kits, Roche Diagnostics, respectively).

### 2.5 Statistical methods

Values are presented as mean ± SD, unless otherwise indicated. The primary outcome of this exploratory study was to compare heart rate variability (HRV) (Low Frequency (LF), High Frequency (HF), LF:HF) at baseline and in response to Ang II challenge at 60 min between postmenopausal women and premenopausal women. The secondary outcome was to compare HRV (LF, HF, LF:HF) at baseline and in response to Ang II challenge at 60 min in premenopausal women in two phases of the menstrual cycle (e.g., high and low estradiol phases). Paired and unpaired *t*‐tests were used to determine significance. HRV measures were log transformed to obtain normalized distributions. ANCOVA models were used to in order to adjust for age and BMI as confounders. Regression models were used to determine associations between serum sex hormone levels (estradiol, progesterone, and testosterone) and HRV measure using age and BMI as confounders. Statistical analyses were performed using SPSS (version 19, IBM, Armonk, NY) and STATA (version 14, StataCorp LP, College Station, TX) with two‐sided statistical significance at *p* < 0.05.

## 3 RESULTS

### 3.1 Baseline characteristics

No participants were living with obesity, hypertension, or diabetes (Table 1). The majority of participants self‐identified as white but there was a significantly lower proportion of postmenopausal women who self‐identified as white compared to premenopausal women (*p* = 0.03). Postmenopausal women were older, and had higher blood pressure and higher total cholesterol compared to premenopausal women, although all values were in the normal range for both groups. Ninety‐two percent of the postmenopausal women were within 10 years of menopause onset and all had experienced natural menopause.

**TABLE 1:** Baseline characteristics of premeopausal and postmenopausal women

Baseline characteristics	All (*n* = 41)	Postmenopausal (*n* = 13)	Premenopausal (*n* = 28)	*p*
Age (years)	40 ± 14	55 ± 4	33 ± 11	< 0.0001
% White	76%	54%	86%	0.03
BMI (kg/m^2^)	25.5 ± 4.2	28.0 ± 5.0	24.3 ± 3.7	0.01
Systolic blood pressure (mmHg)	114 ± 14	123 ± 179	110 ± 9	0.002
Diastolic blood pressure (mmHg)	66 ± 11	71 ± 12	63 ± 71	0.03
Fasting glucose (mmol/L)	4.5 ± 0.5	4.5 ± 0.5	4.5 ± 0.5	0.6
Total cholesterol (mmol/L)	4.2 ± 0.9	4.7 ± 0.96	3.9 ± 0.75	0.005
HDL cholesterol (mmol/L)	1.6 ± 0.3	1.6 ± 0.4	1.6 ± 0.3	0.6
LDL cholesterol (mmol/L)	2.3 ± 0.8	2.6 ± 1.0	2.1 ± 0.64	0.06
Estradiol (pmol/L)				
Mean	315 ± 329	138 ± 242	397 ± 335	0.02
Median [IQR]	162 [415]	42 [62]	341 [382]	
Progesterone (nmol/L)				
Mean	6.7 ± 13.2	1.0 ± 0.4	9.5 ± 15.5	0.06
Median [IQR]	1.3 [1.51]	0.9 [0.3]	1.95 [7.2]	
Testosterone (nmol/L)				
Mean	1.0 ± 0.6	0.7 ± 0.3	1.1 ± 0.6	0.03
Median [IQR]	0.8 [0.6]	0.7 [0.3]	0.9 [1.1]	
Sex hormone binding globulin (nmol/L)	59.2 ± 44.6	51.5 ± 30.7	62.3 ± 49.4	0.5

Sex hormone levels were within the normal range for both postmenopausal and premenopausal women. As anticipated, postmenopausal women had lower levels of estradiol, progesterone, testosterone, and sex hormone binding globulin compared to premenopausal women (Table 1). Eleven premenopausal women were studied in the low and high estradiol phases of the menstrual cycle and sex hormone levels are shown in Table 2.

**TABLE 2:** Sex hormone levels in premenopausal women (low  vs. high estradiol phase)

	Low Estradiol Phase (*n* = 11)	High Estradiol Phase (*n* = 11)
Estradiol		
Mean	298 ± 210*	555 ± 328
Median [IQR]	252 [105]	447 [535]
Progesterone		
Mean	6.7 ± 9.2	11.0 ± 15.8
Median [IQR]	2.0 [6.1]	4.6 [13.0]
Testosterone		
Mean	1.0 ± 0.7	0.9 ± 0.5
Median [IQR]	0.8 [0.9]	1.0 [0.4]

*p < 0.05 versus high estradiol phase.

### 3.2 Postmenopausal versus premenopausal women HRV measures

**FIGURE 1:**
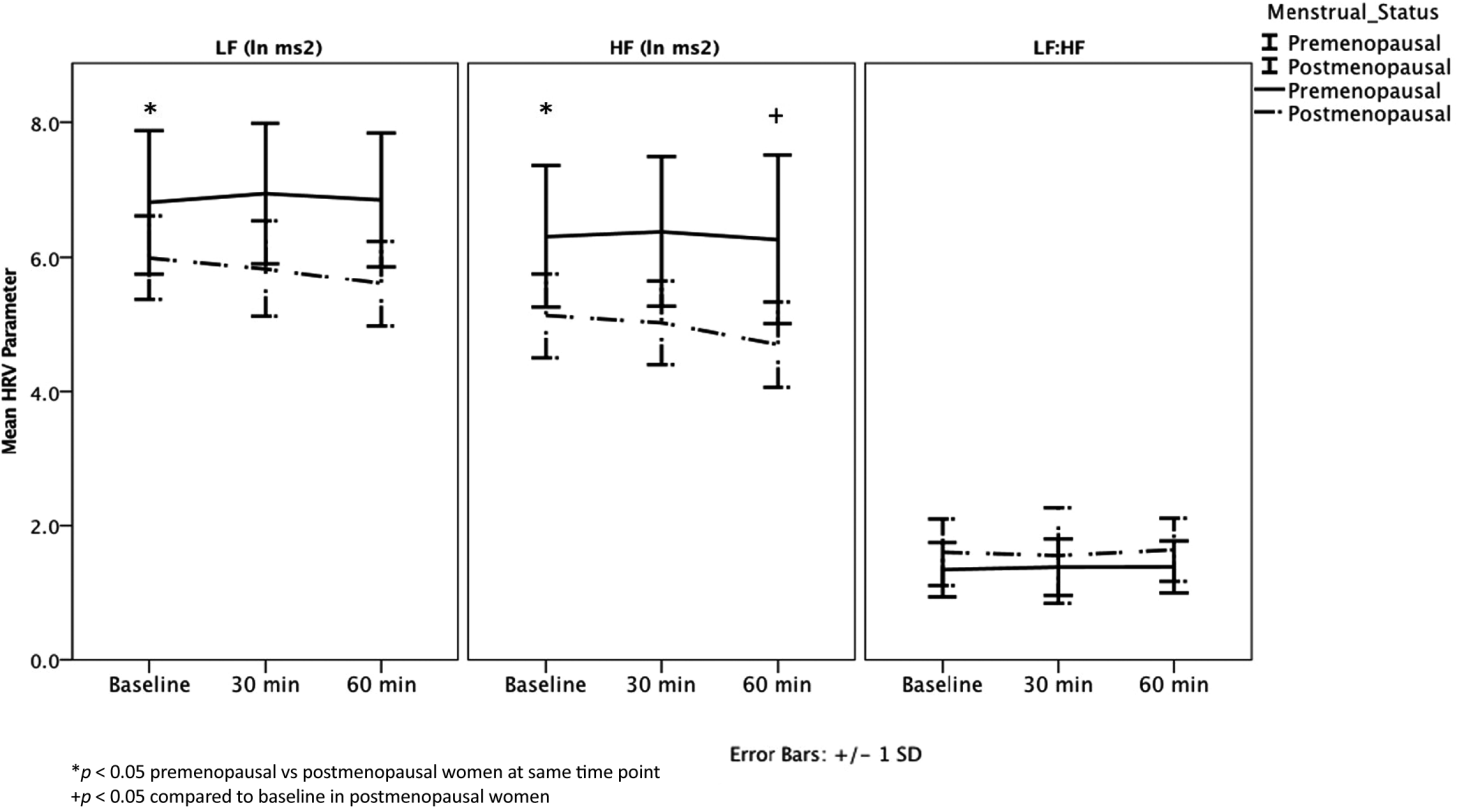
Baseline HRV and HRV response to AngII in premenopausal and postmenopausal women, unadjusted.

All baseline HRV values were within expected ranges for age (Moodithaya & Avadhany, 2009). Postmenopausal women had significantly lower LF (*p* = 0.01) and HF (*p* < 0.001) compared to premenopausal women during the high estradiol phase of the menstrual cycle, though LF:HF did not differ between the groups (*p* = 0.08) (Table 3, Figure 1). = These differences were no longer significant once adjusted for age and BMI (LF *p* = 0.7, HF *p* = 0.3, LF:HF *p* = 0.5).

**TABLE 3:** Baseline HRVand HRV response to AngII infusion in premenopausal versus postmenopausal women

	Postmenopausal (*n* = 13)	Premenopausal (*n* = 28)
*HF (ln ms2)*		
Baseline	5.13 ± 0.63*	6.31 ± 1.05
30 min	5.02 ± 0.62*	6.38 ± 1.11
60 min	4.69 ± 0.63*^,^**	6.26 ± 1.26
*LF (ln ms2)*		
Baseline	6.00 ± 0.62*	6.81 ± 1.06
Response to 3 ng/kg per min Ang II	5.83 ± 0.71*	6.94 ± 1.04
Response to 6 ng/kg per min Ang II	5.60 ± 0.63*	6.85 ± 1.00
*LF:HF*		
Baseline	1.61 ± 0.49	1.35 ± 0.40
Response to 3 ng/kg per min Ang II	1.56 ± 0.71	1.40 ± 0.42
Response to 6 ng/kg per min Ang II	1.64 ± 0.47	1.40 ± 0.38

*p < 0.05 versus premenopausal, unadjusted; **p < 0.05 versus baseline.

**FIGURE 2:**
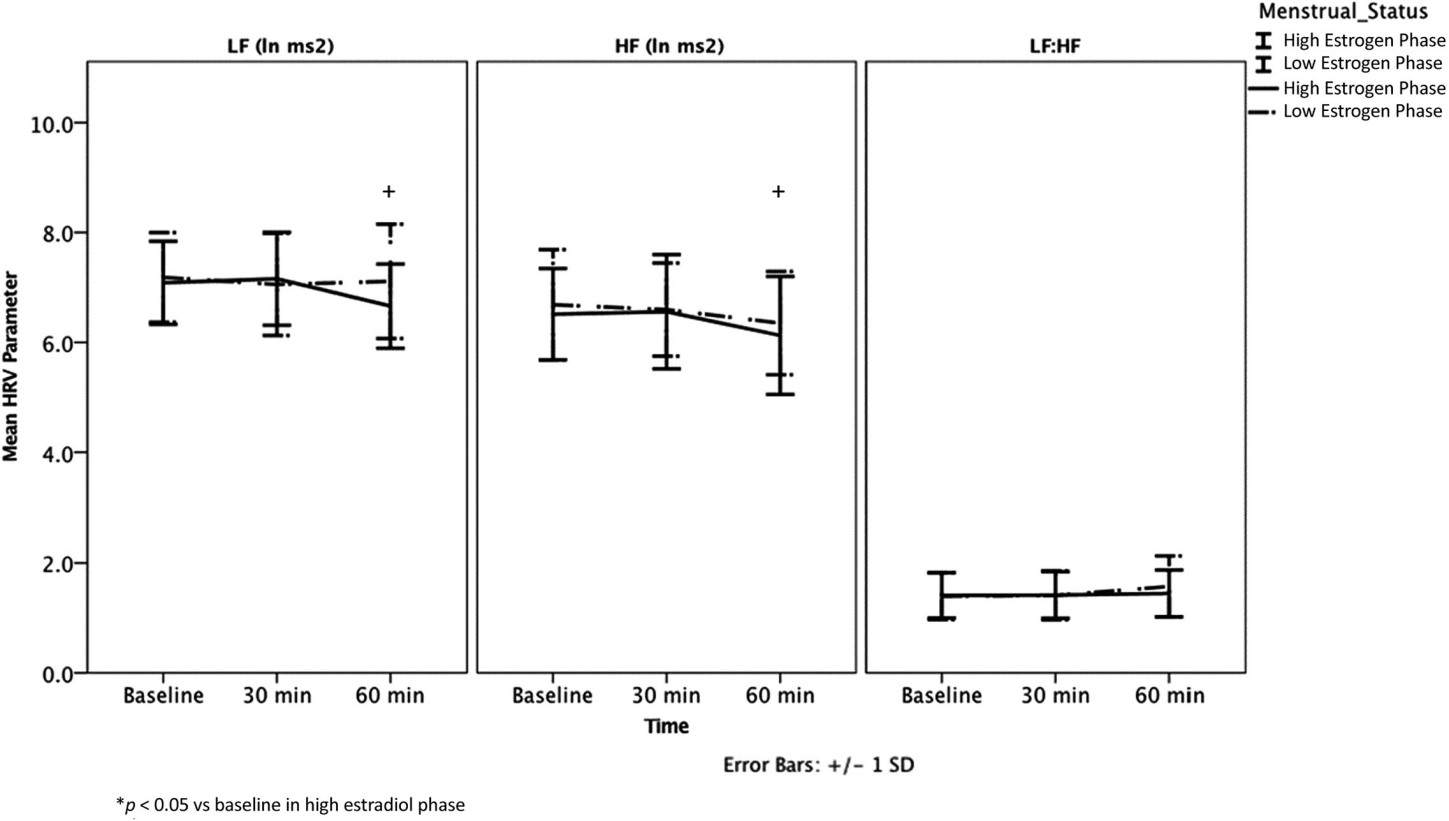
Baseline HRV and HRV response to AngII in the luteal and follicular phase in women.

In response to AngII challenge, postmenopausal women maintained LF (*p* = 0.07, time 60 min vs. baseline) but demonstrated a decrease in cardioprotective HF (ΔHF, –0.43 ± 0.46 ln ms^2^, *p* = 0.005, time 60 min vs. baseline) (Table 3, Figure 1). Overall no change was observed in LF:HF in response to AngII challenge (*p* = 0.8). Conversely, premenopausal women in the high estradiol phase maintained LF (*p* = 0.8), HF (*p* = 0.8) and LF:HF (*p* = 0.6) in response to AngII (Table 3, Figure 2b), though the LF and HF responses to AngII challenge were not statistically different from those observed in the postmenopausal women (LF, *p* = 0.1; HF, *p* = 0.1; LF:HF, *p* = 0.9) with both univariate and multivariate analyses (Table 3).

### 3.3 High versus low estradiol phases of the menstrual cycle

No differences were found in baseline LF (*p* = 0.6), HF (*p* = 0.7), and LF:HF (*p* = 0.9) measures between the high and low estradiol phases of the menstrual cycle in premenopausal women (Table 4, Figure 2). However, in response to AngII challenge, premenopausal women in the high estradiol phase demonstrated both a decrease in LF (ΔLF = −0.07 ± 0.46 ln ms^2^, *p* = 0.048, time 60 min vs. baseline) and HF (ΔHF = −0.33 ± 0.74 ln ms^2^, *p* = 0.048, time 60 min vs. baseline), though no change in overall LF:HF was observed (*p* = 0.86) (Table 4, Figure 2) In contrast, no changes in LF (*p* = 0.6), HF (*p* = 0.2) or LF:HF (*p* = 0.3) (all values time 60 min compared to baseline) in response to AngII challenge were observed in the low estradiol phase.

**TABLE 4:** Baseline HRV and HRV response to AngII infusion in premenopausal women in the low estradiol and high estradiol phase

	Low Estradiol Phase (*n* = 11)	High Estradiol Phase (*n* = 11)
*HF (ln ms2)*		
Baseline	6.69 ± 1.00	6.51 ± 0.83
Response to 3 ng/kg per min Ang II	6.60 ± 0.85	6.56 ± 1.04
Response to 6 ng/kg per min Ang II	6.35 ± 0.94	6.13 ± 1.08*
*LF (ln ms2)*		
Baseline	7.18 ± 0.82	7.08 ± 0.75
Response to 3 ng/kg per min Ang II	7.05 ± 0.93	7.16 ± 0.85
Response to 6 ng/kg per min Ang II	7.11 ± 1.04	6.66 ± 0.77*
*LF:HF*		
Baseline	1.39 ± 0.43	1.41 ± 0.41
Response to 3 ng/kg per min Ang II	1.41 ± 0.45	1.41 ± 0.42
Response to 6 ng/kg per min Ang II	1.57 ± 0.56	1.44 ± 0.43

*p < 0.05 versus baseline in same estradiol phase.

### 3.4 Sex hormones and HRV

No linear associations were observed between serum estradiol, progesterone or testosterone levels and any measure of HRV at baseline or in response to AngII challenge in univariate or multivariate analyses in both postmenopausal and premenopausal groups.

## 4 DISCUSSION

This cross‐sectional study aimed to examine the association between menopausal and menstrual status and estradiol and HRV, a validated marker of CVD risk (Camm et al., 1996) both at baseline and in response to a physiological stressor. Our key findings were as follows: (1) Postmenopausal women demonstrate lower HRV measures compared to premenopausal women, though these differences appear due to age and BMI; (2) postmenopausal women demonstrated a decrease in cardioprotective HF HRV in response to a stressor, while premenopausal women maintained this measure of cardiac autonomic tone in response to AngII; (3) in premenopausal women, baseline HRV measures did not differ between the high and low estradiol phases of the menstrual cycle. However, during the high estradiol phase, women showed a decrease in HRV measures in response to the stressor that was not observed in the low estradiol phase; (4) no association was observed between estradiol level and any measure of HRV, suggesting that while important differences exist in HRV between postmenopausal and premenopausal women, it is unlikely that estradiol is the primary factor.

Previous studies have examined the difference in HRV based on menopausal status (Gökçe et al., 2005; Liu et al., 2003; Mercuro et al., 2000; Moodithaya & Avadhany, 2009; Virtanen et al., 2015; Yang et al., 2013) and the menstrual cycle (Matsumoto et al., 2007; Princi et al., 2005; Sato & Miyake, 2004; Vallejo et al., 2005; Zambotti et al., 2013). However, to the best of our knowledge, this is the first study to investigate differences in the HRV response to a physiological stressor. Liu et al reported differences in baseline HRV between postmenopausal and premenopausal women, though did not adjust for age, which is known to be associated with decreased HRV measures (Liu et al., 2003). This was demonstrated in a study by Moodithaya et al where they reported differences in HF, LF and an increased LF:HF ratio in young postmenopausal women with naturally occurring menopause not on hormone therapy (Moodithaya & Avadhany, 2009), compared to premenopausal women. However, both age and estradiol level appeared to account for these dissimilarities rather than menopausal status. In contrast, Yang and colleagues did not observe differences in these measures by menopausal status at baseline. They did report a decrease in LF in postmenopausal women treated with conjugated equine estradiol alone, but not in those treated with combined estradiol and norethisterone acetate, compared to untreated controls after two months (Yang et al., 2013). In women with surgical menopause, Mercuro et al examined the effect of oophorectomy and treatment with transdermal estradiol on HRV in 14 premenopausal women before and after undergoing oophorectomy compared to control undergoing ovary‐sparing hysterectomy. While baseline HRV levels were similar between the groups, oophorectomy was associated with a decrease in HF and LF with an overall increase in the LF:HF ratio. Estradiol treatment was associated with a non‐significant increase in HF and LF, with an overall decrease in LF:HF. Neves et al compared HRV measures in postmenopausal women not on hormone therapy and postmenopausal women chronically ingesting conjugated equine estrogen with that of a younger group of premenopausal women. Similar HRV measures were observed between the postmenopausal women on estradiol and premenopausal women, but postmenopausal women not on estradiol therapy has lower measures of HF and higher measures of LF and LF:HF. However, age was not taken into account in the analysis. Taken together, the results of these studies suggest that while age remains an important variable when interpreting HRV, factors such as timing since menopausal onset, type of menopause, whether the estradiol exposure is endogenous or exogenous, type of estradiol formulation, route of administration and concomitant use and type of progestin also affect this validated marker of cardiovascular risk. The results of our study suggest that, among women in the early years of natural menopause and who are not taking postmenopausal hormone therapy, HRV measures are similar to those premenopausal women after adjustment for age and BMI.

The association between different stages of the menstrual cycle and HRV has been studied previously, however, the results are conflicting. While some studies report no change in HRV across the menstrual cycle (Matsumoto et al., 2007; Zambotti et al., 2013), others have reported no change in parasympathetic by the menstrual phase but enhanced sympathetic activity during the luteal phase (Sheema et al., 2014; Yazar & Yazıcı, 2016). A recent systematic review and meta‐analysis examining measures of cardiac vagal activity across the menstrual cycle in naturally‐cycling premenopausal females reported a decrease in parasympathetically‐mediated HRV from the low estradiol (follicular) phase to the high estradiol (luteal) phase, but highlighted that differences in the classification of menstrual cycle stage likely contributed to variability in reported study finding (Matsumoto et al., 2007). Additionally, the discrepant results among studies could be due to the presence of premenstrual symptoms, BMI, and dietary intake (Davy et al., 1997; McNeely et al., 2008; Minson et al., 2000), all of which have been shown to influence HRV.

Molecular studies have supported a protective role for estradiol and cardiac autonomic tone in animals (Saleh et al., 2000, 2005; Saleh & Connell, 2003). Estradiol is a steroid hormone that has the ability to cross the blood‐brain barrier (Lee & McEwen, 2001). Furthermore, estradiol receptors are found in the medulla oblongata, which is a potential mechanism through with estradiol could control the autonomic nervous system (Shughrue et al., 1997). Studies have shown that control female Sprague‐Dawley rats have a lower vagal tone in comparison to female Sprague‐Dawley rats that have been given systemic estradiol injections (Saleh et al., 2000; Saleh & Connell, 2003); moreover, local estradiol injections in the localized parts of the brain involved with control of autonomic tone resulted in a similar increase in vagal tone compared to control (Saleh et al., 2000). Additionally, estradiol injections in the autonomic regulatory parts of the brain in ovariectomized female rats led to a decrease in sympathetic activity (Saleh et al., 2000), highlighting possible mechanisms for the role of estradiol in the maintenance of cardiac autonomic tone.

In our study, endogenous estradiol levels were not associated with HRV, either at baseline or in response to AngII challenge in postmenopausal or premenopausal women. This may reflect differences between endogenous and exogenous estradiol exposure. However, postmenopausal women demonstrated a decrease in cardioprotective HF in response to AngII compared to premenopausal women in the high estradiol phase of the menstrual cycle. In contrast, premenopausal women in the high estradiol phase of the menstrual cycle demonstrated a decrease in both HF and LF in response to AngII challenge, but not in the low estradiol phase. We speculate that these responses represent differences between chronically low estradiol, as observed in menopause, and acute changes in estradiol, as seen in the menstrual cycle.

Similar to our findings, previous studies have highlighted differing responses to stimuli during the high estradiol (luteal) and low estradiol (follicular) phases of the menstrual cycle (Chidambaram et al., 2002). Circulating RAAS components are highest when estradiol levels peak during the luteal phase, suggesting that estradiol is involved in the activation of the RAAS (Davis et al., 2017). However, despite increased circulating RAAS components during the high estradiol phase, women in the luteal phase were unable to to maintain blood pressure in response to lower body negative pressure, though maintained BP in response to the same stressor during the low estradiol phase. Moreover, women in this study demonstrated similar responses to angiotensin receptor blockade and AngII challenge, suggesting that the changes in estradiol throughout the cycle mediate tissue‐specific effects (Chidambaram et al., 2002). Other studies have suggested a role for differences in progesterone levels during the menstrual cycle as progesterone, but not estradiol, levels were associated with the aldosterone response to AngII challenge in premenopausal women (Szmuilowicz et al., 2006). Similarly, progesterone, but not estradiol, levels were associated with the vascular response to AngII challenge in hypertensive postmenopausal women (Szmuilowicz et al., 2007). Previous studies have indicated that testosterone (Ermis et al., 2010) and progesterone (Auger & De Vries, 2002; Genazzani et al., 2000) are also associated with HRV and autonomic tone, suggesting an important role of the hormonal milieu over the exposure to any individual sex hormone. While we did not observe an association between sex hormones and measures of HRV, this may reflect a narrow range of sex hormone levels measured in our study population.

This study has both strengths and limitations. First, given HRV can be influenced by lifestyle factors such as diet, obesity, and smoking (Karason et al., 1999; Kobayashi et al., 2005; Piccirillo et al., 1996), we included only healthy subjects who did not smoke, were not obese, and were in high‐salt balance state reflective of the typical Western diet (Brown et al., 2009). Second, while 24 hour Holter ECG monitoring is considered the gold standard for measurement of HRV, the use of short term ECG recordings for heart rate variability has been previously shown to have excellent correlation to 24 h measurement (La Rovere et al., 2003). Third, our sample size was limited; however, the study of a homogenous population of healthy individuals without comorbidities or exogenous estradiol use on a high salt diet in a controlled lab environment minimized the effect of confounders. Fourth, the participants in this study had sex hormone levels in the normal range, which may limit the power to detect a significant difference. However, this eliminates the possibility of a spectrum bias within the study and makes it more representative of a healthy population. Finally, we would like to highlight the drawback of multiple comparisons within our study and our results should be considered with this limitation in mind; however, this study is exploratory in nature, and we believe these findings are important to further understand the underlying physiology of cardiovascular risk in women, a largely understudied population. Notably, recent literature highlights the paucity of preclinical CV research in both female animal models and human populations, underscoring the novelty and importance of the current study (Saxena et al., 2012).

The findings of our study suggest that age, but not endogenous estradiol, is primarily responsible for the decrease in measures of HRV and unfavorable response to an acute stressor observed in postmenopausal women compared to premenopausal women. Conversely, important differences in the HRV response to AngII were observed in the high estradiol phase of the menstrual cycle compared to the low estradiol phase, suggesting that acute changes in sex hormone levels in premenopausal women play a role in cardiovascular risk (Saxena et al., 2012).

Globally, cardiovascular disease is the leading cause of death in women, highlighting the need to urgently identify novel female‐specific factors that may influence risk. Further studies are required to elucidate the role of sex hormones, and specifically estradiol, in modulating cardiovascular risk in women.

## AUTHOR CONTRIBUTIONS

Sharanya Ramesh, Matthew T. James, Stephen B. Wilton, Jayna M. Holroyd‐Leduc, and Sofia B. Ahmed conceived the research idea and designed the study; Darlene Y Sola conducted the study; Sharanya Ramesh and Sofia B. Ahmed performed statistical analyses; Sharanya Ramesh and Sofia B. Ahmed drafted the manuscript and Matthew T. James, Stephen B. Wilton, Jayna M. Holroyd‐Leduc, and Darlene Y Sola edited the draft. Each author provided important inputs during the manuscript drafting and revision.
